# Investigating trends in those who experience menstrual bleeding changes after SARS-CoV-2 vaccination

**DOI:** 10.1126/sciadv.abm7201

**Published:** 2022-07-15

**Authors:** Katharine M. N. Lee, Eleanor J. Junkins, Chongliang Luo, Urooba A. Fatima, Maria L. Cox, Kathryn B. H. Clancy

**Affiliations:** ^1^ Division of Public Health Sciences, Department of Surgery, Washington University in St. Louis School of Medicine, St. Louis, MO, USA.; ^2^ Department of Anthropology, University of Illinois at Urbana-Champaign, Champaign, IL, USA.; ^3^ Studies of Women, Gender, and Sexuality, Harvard University, Cambridge, MA, USA.; ^4^ Department of Psychology, University of Illinois at Urbana-Champaign, Champaign, IL, USA.; ^5^ Program in Ecology, Evolution, and Conservation, University of Illinois at Urbana-Champaign, Champaign, IL, USA.; ^6^ Beckman Institute of Advanced Science and Technology, University of Illinois at Urbana-Champaign, Champaign, IL, USA.; ^7^ Institute for Genomic Biology, University of Illinois at Urbana-Champaign, Champaign, IL, USA.

## Abstract

Early in 2021, many people began sharing that they experienced unexpected menstrual bleeding after SARS-CoV-2 inoculation. We investigated this emerging phenomenon of changed menstrual bleeding patterns among a convenience sample of currently and formerly menstruating people using a web-based survey. In this sample, 42% of people with regular menstrual cycles bled more heavily than usual, while 44% reported no change after being vaccinated. Among respondents who typically do not menstruate, 71% of people on long-acting reversible contraceptives, 39% of people on gender-affirming hormones, and 66% of postmenopausal people reported breakthrough bleeding. We found that increased/breakthrough bleeding was significantly associated with age, systemic vaccine side effects (fever and/or fatigue), history of pregnancy or birth, and ethnicity. Generally, changes to menstrual bleeding are not uncommon or dangerous, yet attention to these experiences is necessary to build trust in medicine.

## Teaser

Increased bleeding can occur after SARS-CoV-2 vaccines; this study investigates patterns in who experiences these changes.

## INTRODUCTION

Menstruating and formerly menstruating people began sharing that they experienced unexpected bleeding after being administered a severe acute respiratory syndrome coronavirus 2 (SARS-CoV-2) vaccine in early 2021. Vaccine trial protocols do not typically monitor for major adverse events for more than 7 days, and additional follow-up communications do not inquire about menstrual cycles or bleeding. Therefore, manufacturers had no way of addressing the extent to which this observation was a coincidence or a potential side effect of the vaccines. In media coverage, medical doctors and public health experts hastened to say that there was “no biological mechanism” or “no data” to support a relationship between vaccine administration and menstrual changes. In other cases, experts declared that these changes were more likely a result of “stress” (*1*–*4*).

Unfortunately, dismissal by medical experts fueled greater concerns, as both vaccine-hesitant and anti-vaccine individuals and organizations conflated the possibility of short-term menstrual changes with long-term harms to fertility. Pundits, politicians, religious leaders, and wellness influencers worked the oft-used framing of protecting women to advise against the vaccine (*5*–*9*). As the SARS-CoV-2 vaccine became available to adolescents, calls to understand the menstrual changes associated with the vaccine increased as parents felt that they were weighing their child’s pubertal development and future fertility against their risk of becoming sick with coronavirus disease 2019 (COVID-19) (*10*, *11*).

There are multiple plausible biological mechanisms to explain a relationship between an acute immune challenge like a vaccine (*12*), its corresponding and well-known systemic effects on hemostasis and inflammation (*13*), and menstrual repair mechanisms of the uterus (*14*–*17*). The uterine reproductive system is flexible and adaptable in the face of stressors to weather short-term challenges in a way that leaves long-term fertility intact (*18*, *19*). We know that running a marathon may influence hormone concentrations in the short term while not rendering that person infertile (*20*), that short-term calorie restriction that results in a loss of menstrual cycling can be overcome by resuming normal feeding (*21*), that inflammation influences ovarian hormones (*22*–*24*), and that psychosocial stressors can correspond to cycle irregularity and yet resilience can buffer one from these harms (*25*–*27*). Less severe, short-term stressors can and do influence menstrual cycling and menstruation, and this has been established over 40 years of cycle research (*19*, *20*, *28*–*30*). This work has also established that while sustained early stressors can influence adult hormone concentrations, short-term stressors resolve and do not produce long-term effects (*31*). The immune response invoked by a vaccine is quite different from the sustained immune assault of COVID-19 itself: Studies and anecdotal reports are already demonstrating that menstrual function may be disrupted long term, particularly in those with long COVID (*32*–*35*).

Vaccines function by mobilizing the immune system to protect from disease if exposure occurs. This immune activation is important, although it may also produce a cascade of other localized (e.g., soreness at injection site) or systemic (e.g., fatigue and/or fever) inflammatory responses. Studies that assess the direct effect of vaccination on the menstrual cycle are few and far between. A study from 1913 identified that the typhoid vaccine was associated with menstrual irregularities, which included missed, late, and early menstruation; discomfort; and heavy bleeding in more than half of their female sample (*36*). Hepatitis B studies have also indicated that menstruation could be altered (*37*), and a human papillomavirus postmarket safety study found that more than a quarter of participants reported menstrual irregularity (*38*), although ovarian insufficiency, a type of reduced fertility analyzed because of case reports, is not associated with this vaccine (*39*). The speed and coverage of the current COVID-19 pandemic and vaccination campaign may have inadvertently highlighted a previously underrecognized side effect of especially immunogenic vaccines administered in adulthood, which is that systemic inflammatory responses may in some individuals invoke downstream responses in target organs such as the uterus.

The question of whether and when the particular acute immune challenge of the current SARS-CoV-2 vaccines affects menstrual cycling or menstruation is an emerging one with limitations on study design. Given the vaccines’ overall established safety generally (*40*–*42*) and in relation to fertility and pregnancy (*43*–*48*), and the multiple waves of viral spread and variant emergence the world has endured with this deadly pandemic, we opted for an observational and retrospective study design of vaccinated people rather than a prospective design with a control or crossover group of unvaccinated individuals. In early anecdotal reports of menstrual cycle experiences, the nature and breadth of the cycle changes were unclear: Among those experiencing side effects, were people experiencing earlier, later, heavier, or lighter periods? Were other menstrual cycle phenomena also altered, like midcycle and premenstrual experiences? Were formerly menstruating people (e.g., those on menstrual suppression therapies or postmenopausal people) affected?

For this reason, we established an emergent, exploratory, mixed-methods survey instrument intended to capture a wide range of responses from current and formerly menstruating adults. Here, we share results from our first round of analyses (*N* = 39,129), as well as the ways that this early exploration has made it possible to establish the parameters of the phenomenon of postvaccine menstrual change. We focus on findings related to menstrual bleeding (in people who menstruate regularly) or breakthrough bleeding (in people who do not currently menstruate) from the first 3 months of data collection to provide a description of trends to clinicians and the public alike. Specifically, we sought to address the following research questions: (i) What is the range of menstrual bleeding changes reported by regularly menstruating respondents after being administered the SARS-CoV-2 vaccine? (ii) To what extent are nonmenstruating respondents reporting breakthrough bleeding after being administered the SARS-CoV-2 vaccine? (iii) Are there trends among those with a changed bleeding pattern to help determine proximate mechanisms acting on the uterus?

Answers derived from this convenience observational sample can help shape the narrative around the nature of short-term menstrual changes, help clinicians working with vaccine-hesitant patients, and develop the necessary, on-the-ground data on this previously unidentified phenomenon to design future prospective, mechanistic studies on the relationship between vaccine immune responses and menstrual repair. Projects that take the time to establish trends and listen to respondents are important first steps to understanding details of emerging health concerns (*49*).

## RESULTS

### Demographics and summary statistics

**Fig. 1.**
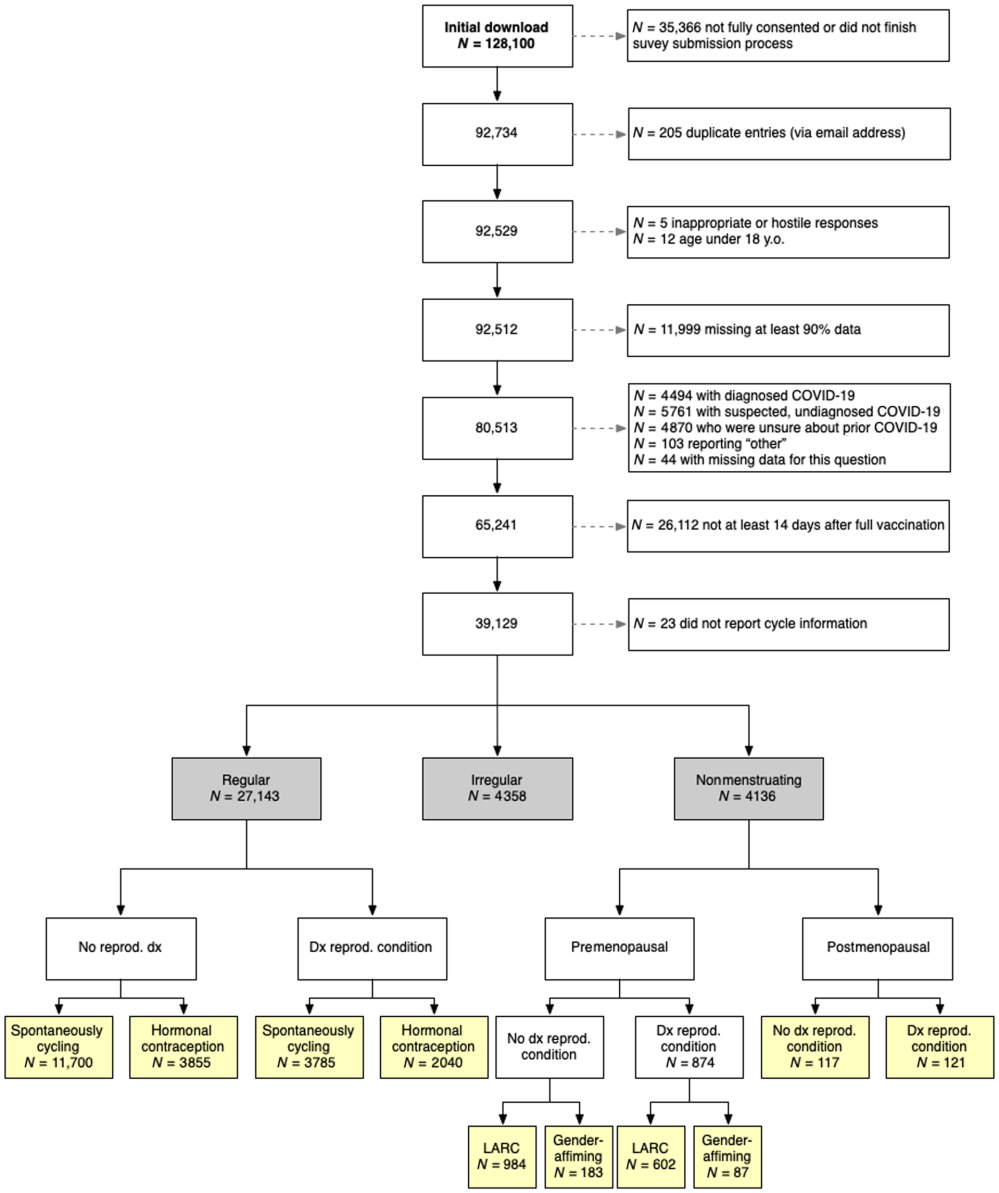
Flowchart of data cleaning and aggregation. Note that totals in the yellow boxes do not add up to the numbers in the gray boxes due to uncertain menopause stage (*n* = 1522), currently or recently lactating (*n* = 2498), having had a hysterectomy (*n* = 43), discrepant responses (e.g., self-reported period details did not align with self-reported menstrual group), and the divisions made to the samples. Further details can be found in the Supplementary Materials. dx, diagnosis.

After data cleaning and aggregation of the first 3 months of data (Fig. 1), respondents in our sample (*N* = 39,129) were between 18 and 80 years old (median = 33 years; *M*_age_ = 34.22 years, SD = 9.18). All participants were fully vaccinated (at least 14 days after one or two required doses as this was before boosters) and had not contracted COVID-19 (diagnosed or suspected). This sample included 35,572 (90.9%) woman-only identifying and 3557 (9.1%) gender-diverse respondents; 32,983 (84.3%) white-only identifying and 6146 (15.7%) racially diverse respondents; and 31,134 (79.6%) non-Hispanic or non-Latinx and 7995 (20.4%) Hispanic, Latinx, or other respondents (summary demographics in Table 1; more details in table S1).

**Table 1.** Sample summary information. Demographics and sample background in life stages corresponding to later sample restrictions (premenopausal, 18 to 45; menopause transition or perimenopause, 46 to 54; postmenopause, 55+). Note that <10 was used for any cells with fewer than 10 individuals. Ages are binned on the basis of approximate life stages and the sample restrictions. *, 23 respondents had two doses of the vaccine but did not specify the vaccine type.

	**Total (*N* = 39,129)**		**18–24 (*N* = 6332)**		**25–34 (*N* = 14,797)**		**35–45 (*N* = 13,096)**		**46–54 (*N* = 4304)**		**55+ (*N* = 600)**	
Age	34.22 (9.18)		21.69 (1.85)		29.63 (2.84)		39.43 (3.10)		49.10 (2.38)		59.34 (4.94)	
Vaccine*												
Pfizer	21,620	55.3%	3646	57.6%	8246	55.7%	7135	54.5%	2287	53.1%	306	51.0%
Moderna	13,001	33.2%	1916	30.3%	4898	33.1%	4521	34.5%	1448	33.6%	218	36.3%
Johnson & Johnson	3469	8.9%	634	10.0%	1260	8.5%	1126	8.6%	406	9.4%	43	7.2%
Other	1016	2.6%	133	2.1%	388	2.6%	304	2.3%	159	3.7%	32	5.3%
Gender												
Identifies woman-only	35,572	90.9%	4535	71.6%	13,449	90.9%	12,751	97.4%	4245	98.6%	592	98.7%
Gender diverse	3557	9.1%	1797	28.4%	1348	9.1%	345	2.6%	59	1.4%	<10	–
Race												
Identifies white-only	32,983	84.3%	4978	78.6%	12,336	83.4%	11,393	87.0%	3743	87.0%	533	88.8%
Racially diverse	6146	15.7%	1354	21.4%	2461	16.6%	1703	13.0%	561	13.0%	67	11.2%
Ethnicity												
Non-Hispanic/ Latinx	31,134	79.6%	4896	77.3%	11,791	79.7%	10,597	80.9%	3409	79.2%	441	73.5%
Hispanic/Latinx or other	7995	20.4%	1436	22.7%	3006	20.3%	2499	19.1%	895	20.8%	159	26.5%
IUDs												
Hormonal	3694	9.4%	540	8.5%	1725	11.7%	1141	8.7%	274	6.4%	14	2.3%
Copper/ nonhormonal	1533	3.9%	157	2.5%	722	4.9%	537	4.1%	112	2.6%	<10	0.8%
Unknown	47	0.1%	<10	0.1%	17	0.1%	16	0.1%	<10	0.1%	<10	0.3%
Hormonal treatments												
Hormonal contraceptive	7438	19.0%	1980	31.3%	3588	24.2%	1583	12.1%	277	6.4%	10	1.7%
Other hormonal treatments	2980	7.6%	377	6.0%	867	5.9%	1082	8.3%	518	12.0%	136	22.7%
Cycle regularity												
Regular	28,811	73.6%	4418	69.8%	11,513	77.8%	11,167	85.3%	2662	61.8%	51	8.5%
Irregular	4768	12.2%	1206	19.0%	1903	12.9%	989	7.6%	632	14.7%	38	6.3%
Nonmenstruating	4525	11.6%	707	11.2%	1377	9.3%	931	7.1%	1003	23.3%	507	84.5%
Medical history												
Past pregnancy	16,859	43.1%	167	2.6%	3980	26.9%	8841	67.5%	3403	79.1%	468	78.0%
Parity	14,579	37.3%	66	1.0%	3049	20.6%	7939	60.6%	3099	72.0%	426	71.0%
Reproductive conditions												
Menorrhagia or heavy bleeding	6864	17.5%	876	13.8%	2123	14.3%	2529	19.3%	1119	26.0%	217	36.2%
Endometriosis	1735	4.4%	142	2.2%	536	3.6%	749	5.7%	266	6.2%	42	7.0%
PCOS	3238	8.3%	391	6.2%	1325	9.0%	1194	9.1%	293	6.8%	35	5.8%
Fibroids	2449	6.3%	32	0.5%	339	2.3%	1151	8.8%	774	18.0%	153	25.5%
Adenomyosis	277	0.7%	11	0.2%	57	0.4%	136	1.0%	64	1.5%	<10	–
Other	2612	6.7%	351	5.5%	956	6.5%	963	7.4%	292	6.8%	50	8.3%

**Fig. 2.**
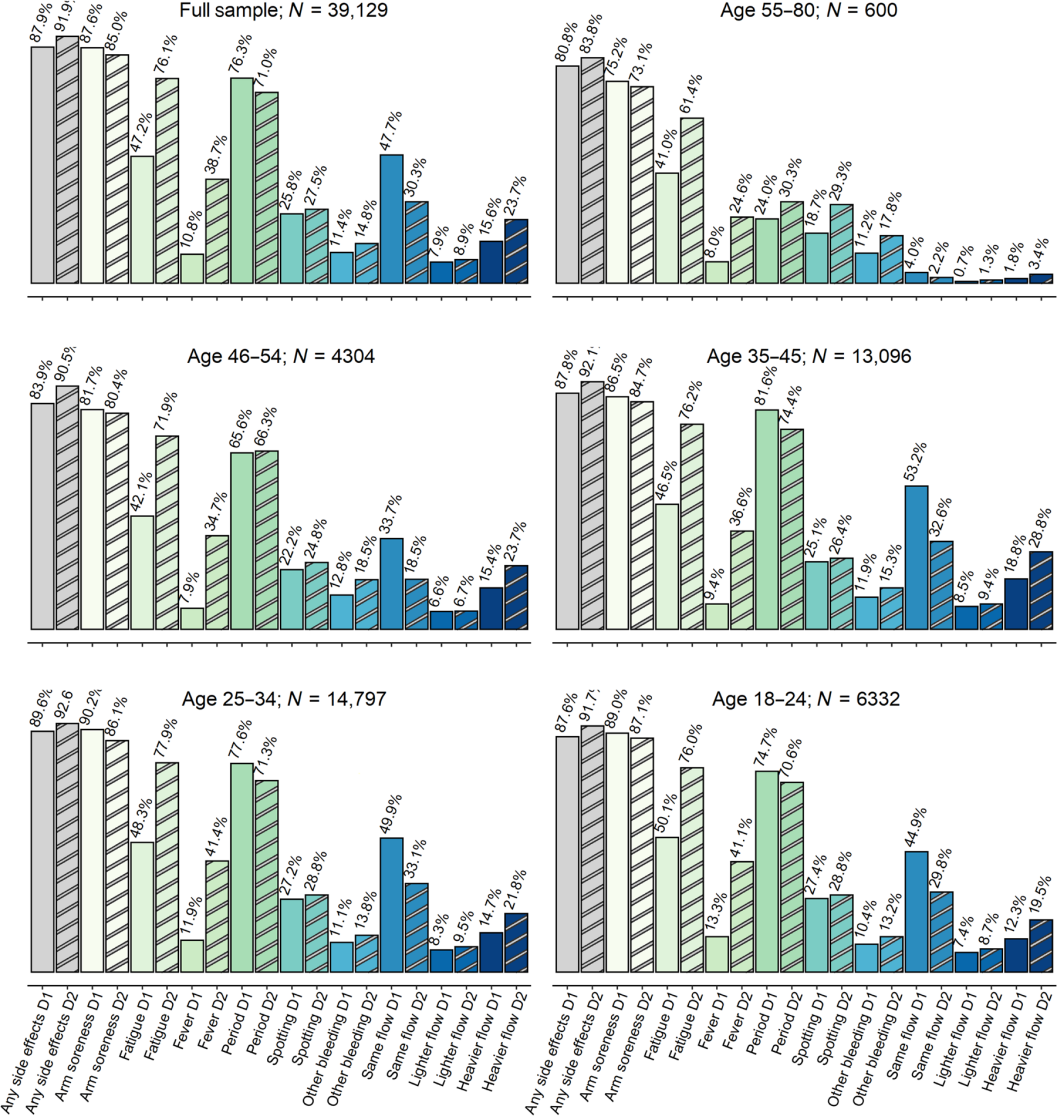
Descriptive statistics of the full sample (dose 1 displayed in solid bars and dose 2 displayed in striped bars). The most salient vaccine and menstrual side effects pertaining to the analysis are presented here. The sample sizes of dose 2 variables decrease because of those who received the one-dose Johnson & Johnson vaccine. The respective samples become as follows: full, *N* = 35,660; age 18 to 24, *N* = 5698; age 25 to 34, *N* = 13,537; age 35 to 45, *N* = 11,970; age 46 to 54, *N* = 3898; age 55 to 80, *N* = 557.

Respondents were vaccinated with Pfizer (*N* = 21,620), Moderna, (*N* = 13,001), AstraZeneca (*N* = 751), Johnson & Johnson (*N* = 3469), Novavax (*N* = 61), or other (*N* = 204) vaccines, with 23 not reporting vaccine type. Self-report of localized vaccine side effects (soreness at injection site) after the first dose and second dose were 87.6 and 77.4%, respectively, when combined across all vaccine types. After the first and second dose, 54.3 and 74.6% of respondents (respectively) report experiencing at least one of the common systemic vaccine side effects (headache, nausea, fever, and/or fatigue). Of those that reported systemic vaccine side effects, 40.6% experienced systemic effects after both doses. Vaccine symptoms, period flow changes, period symptoms, and timing of period symptoms reported by study respondents are presented by age categories (Fig. 2; detailed reporting by vaccine type in table S2). The Johnson & Johnson vaccine, being the only single-dose vaccine at the time of survey, was excluded from later analyses.

### Reported menstrual changes in regularly menstruating people

Respondents reported noticing changes to their period 1 to 7 days after vaccines (dose 1: 31.4%; dose 2: 37.0%), 8 to 14 days after vaccines (dose 1: 25.9%; dose 2: 23.6%), or more than 14 days after receiving their vaccines (dose 1: 29.9%; dose 2: 26.8%), with the rest of respondents reporting that they were menstruating when they received the vaccine (dose 1: 12.7%; dose 2: 12.5%). In total, 42.1% reported heavier menstrual flow after vaccines, 14.3% reported not heavier (characterized by a mix of lighter or no change) menstrual flow, and 43.6% reported no change to flow after vaccines.

#### Associations with a heavier postvaccine menstrual flow

**Fig. 3.**
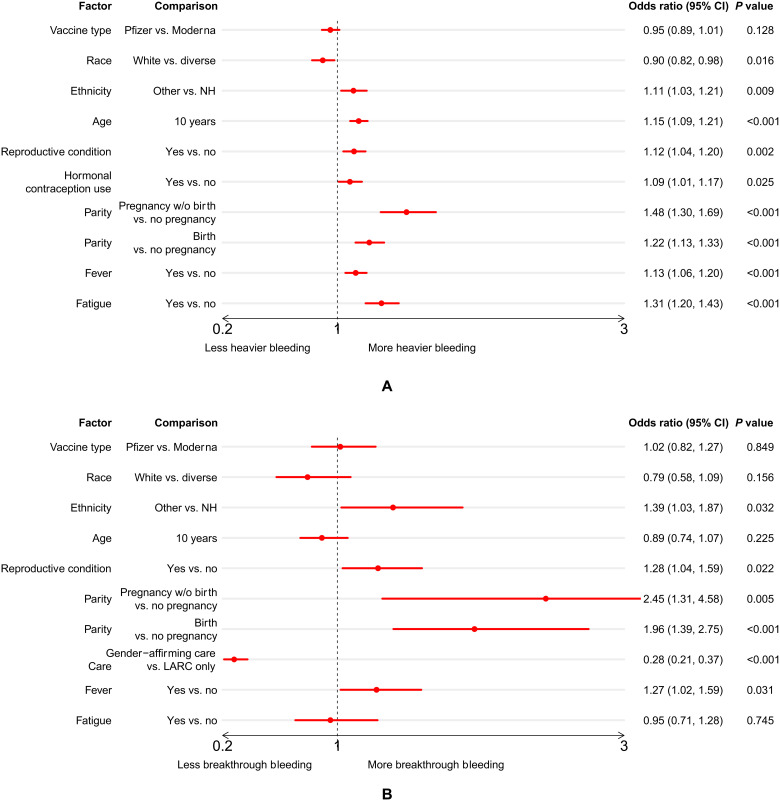
Results of multivariate regressions. Multivariate logistic regression of (**A**) heavier flow in the regularly menstruating group (*N* = 17,113, after removing those respondents with vaccines other than Pfizer or Moderna, or missing parity history or flow change) and (**B**) breakthrough bleeding in the nonmenstruating premenopausal group (*N* = 1771, after removing those respondents with vaccines other than Pfizer or Moderna, or missing parity history) after either dose of the vaccine. The graph presents the ratio of the odds of heavy bleeding occurring in the first group of the comparison versus the second group (except for age, which is in 10-year increments). If the odds ratio is greater than 1, the first group in the comparison has higher risk of experiencing heavier bleeding (or breakthrough bleeding). NH, not Hispanic/Latinx; CI, confidence interval.

Following the univariate tests for association (table S6), we fit a multivariate logistic regression model to explore the relationship between heavier menstrual bleeding after vaccination and several factors: vaccine type, demographic factors, reproductive history, hormonal contraception use, and systemic vaccine response (Fig. 3A). Our main findings were that a heavier menstrual flow was more likely for those respondents who were of non-white race, were Hispanic/Latinx, were older, had a diagnosed reproductive condition, used hormonal contraception, had been pregnant in the past (whether or not they had given birth), were parous, or experienced fever or fatigue after vaccination. The comparison between those who have given birth and those who have not is conditioned on having been pregnant: The combination of a reproductive history that includes being pregnant but not giving birth in the past is associated with the highest risk of heavier flow, although we note that parity is also associated with heavier flow. We note that vaccine type, race, and use of hormonal contraceptives have odds ratios and 95% confidence intervals very close to or overlapping with 1 in combination with a relatively high *P* value, suggesting that they have negligible or relatively small effects in this model.

#### Reproductive conditions

**Fig. 4.**
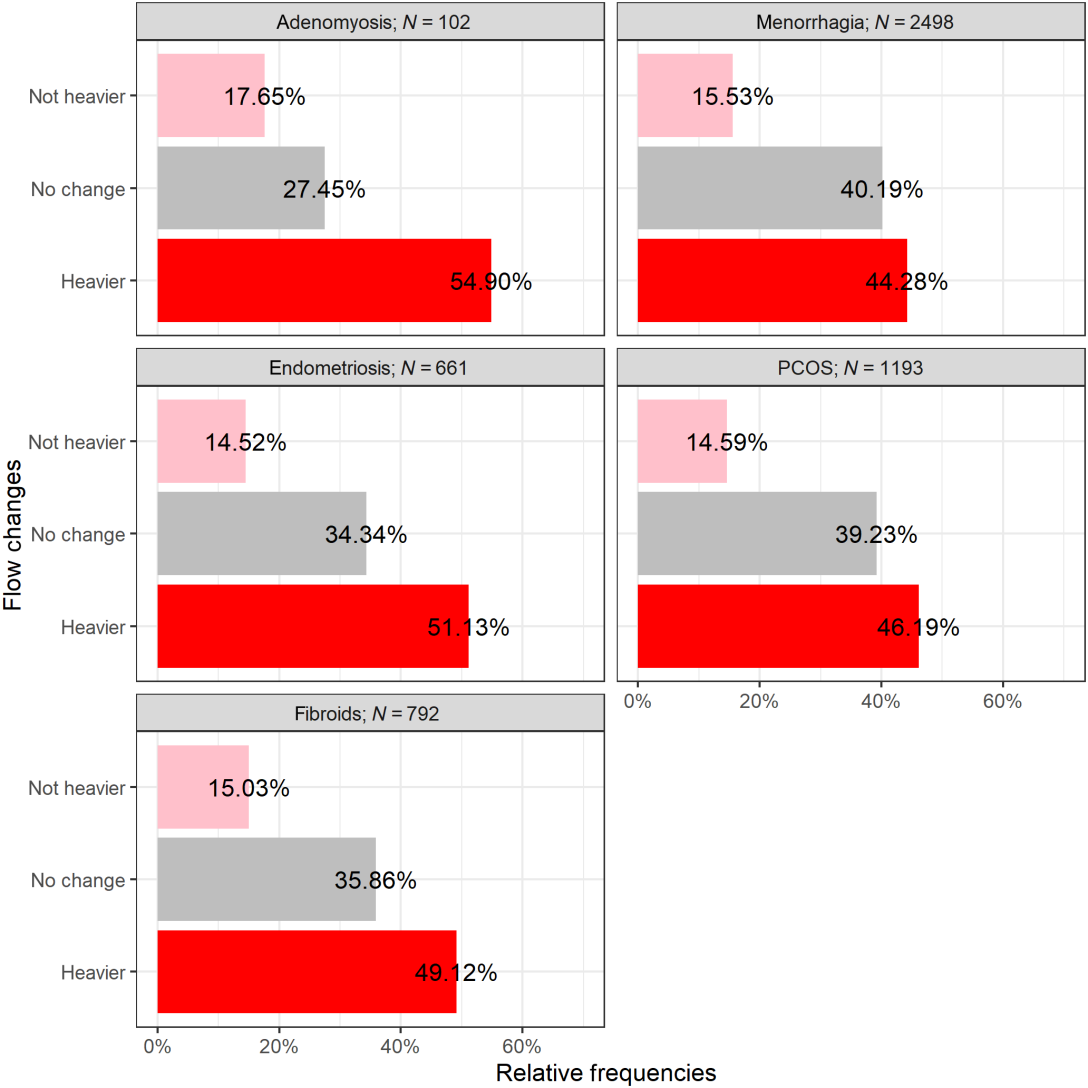
Menstrual flow changes in regularly cycling individuals with diagnosed reproductive conditions. Displayed on the *x* axis is the percentage of individuals reporting each flow change condition (*y* axis).

We additionally examined the relationship of specific reproductive conditions often associated with altered menstrual bleeding by comparing respondents with diagnosed conditions to respondents with no reported reproductive conditions (Fig. 4). A higher proportion of respondents with endometriosis (51.1%), menorrhagia (44.3%), fibroids (49.1%), polycystic ovarian syndrome (PCOS) (46.2%), and adenomyosis (54.9%) reported experiencing a heavier menstrual flow after vaccine than the respondents without diagnosed reproductive conditions (40.9%). Odds ratios and chi-square results for these groups are in table S7.

### Reported breakthrough bleeding in nonmenstruating respondents

**Fig. 5.**
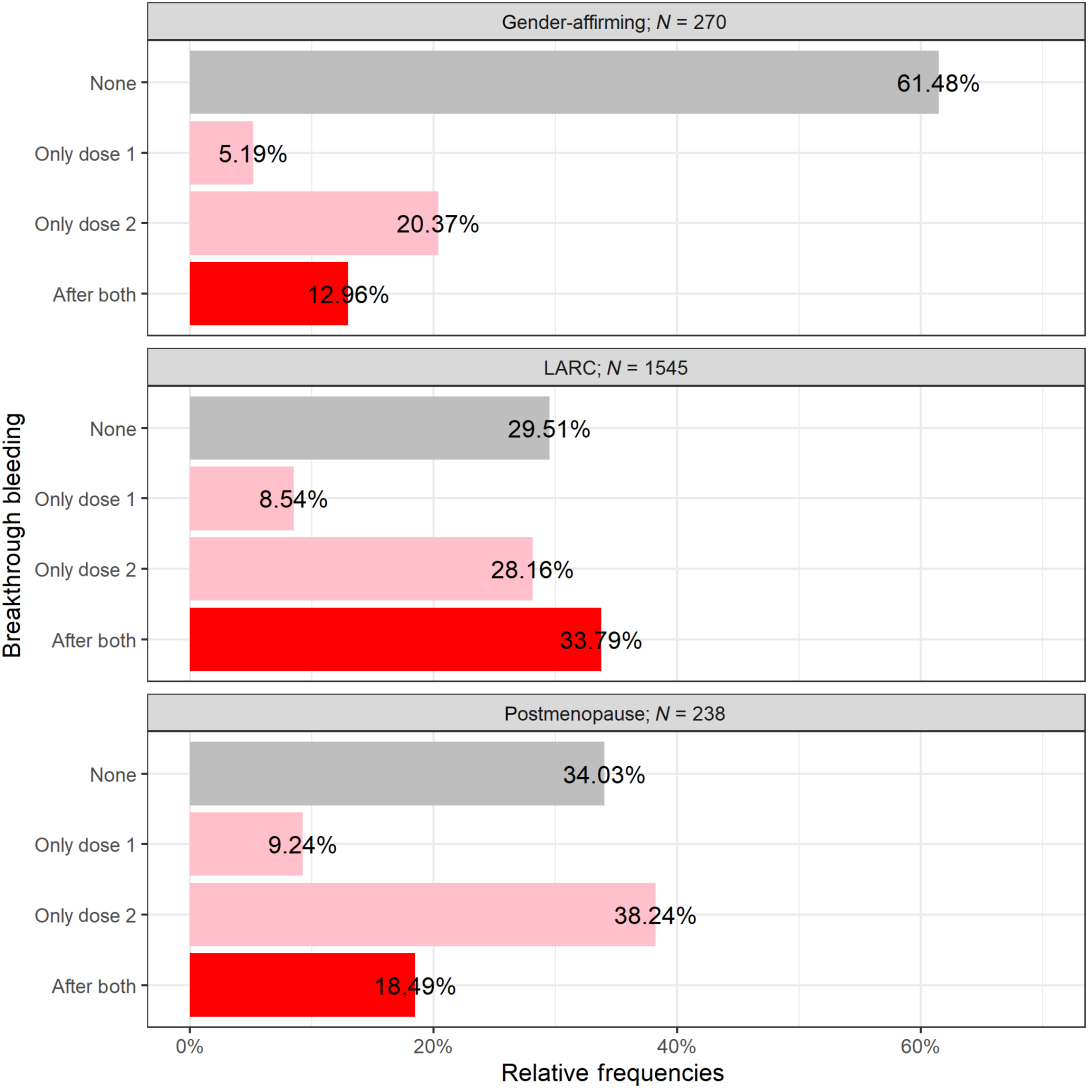
Breakthrough bleeding in nonmenstruating individuals. Displayed on the *x* axis is the percentage of individuals reporting breakthrough bleeding after both doses, only following dose 2, only following dose 1, or no breakthrough bleeding during vaccination time (*y* axis).

Nonmenstruating people consisted of two groups: premenopausal people [using long-acting reversible contraceptives (LARCs) and/or continuous hormonal contraceptives and/or gender-affirming treatment that eliminates menstruation] and postmenopausal people over the age of 55 who had not bled for at least 12 months (before SARS-CoV-2 vaccination). Among nonmenstruating, premenopausal respondents (*N* = 1815) on hormonal treatments, a majority of this sample (65.7%) experienced breakthrough bleeding after a vaccine, although this was significantly different between respondents using only LARC (70.5%) and respondents using gender-affirming care (38.5%). Among postmenopausal people who were not on any hormonal treatments (*N* = 238), breakthrough bleeding was reported by 66.0% of respondents (Fig. 5).

#### Associations with breakthrough bleeding in premenopausal respondents

A multivariate logistic regression of breakthrough bleeding in the nonmenstruating premenopausal group after either dose of the vaccine was fitted. The result (Fig. 3B) shows an increased chance of breakthrough bleeding for those respondents who were Hispanic/Latinx, had been pregnant in the past but had not given birth, had a diagnosed reproductive condition, were on LARC only, or experienced fever after vaccination.

#### Associations with breakthrough bleeding in postmenopausal respondents

Age was significantly different between those that experienced breakthrough bleeding occurrence or not [*t*(147.99) = −2.255, *P* = 0.026], with postmenopausal people who experienced breakthrough bleeding being slightly younger (*M* = 59.8 years) than those who did not (*M =* 61.4 years). Ethnicity was associated with breakthrough bleeding, with non-Hispanic/Latinx respondents being less likely to report breakthrough bleeding. There was no significant difference in rate of occurrence of breakthrough bleeding by vaccine type, systemic side effects of fever or fatigue, or reproductive history of past pregnancy or parity (Table 2).

**Table 2.** Breakthrough bleeding in postmenopausal people. Vaccine and medical history related to breakthrough bleeding across postmenopausal respondents. Associations with breakthrough bleeding were investigated on the binary outcome. Alpha thresholds used for postmenopause were *P* < 0.05.

	**Postmenopause**							
	**Breakthrough bleeding**			**Chi-square results**			**Effect size**	
	Yes	No	*N*	df	χ^2^	*P*	φ_c_	φ_c_ 95% CI
Vaccine type								
Pfizer	66.1%	33.9%	124					
Moderna	60.4%	39.6%	96	1	0.54	0.464	0.059	[0.004, 0.198]
Vaccine symptoms								
Fever	67.7%	32.3%	62					
No fever	65.3%	34.7%	176	1	0.04	0.851	0.022	[0.002, 0.147]
Fatigue	67.1%	32.9%	164					
No fatigue	63.5%	36.5%	74	1	0.15	0.698	0.035	[0.002, 0.168]
Medical history								
Parous	64.9%	35.1%	168					
Not parous	68.6%	31.4%	70	1	0.16	0.691	0.035	[0.003, 0.160]
Pregnant	64.7%	35.3%	187					
Not pregnant	70.6%	29.4%	51	1	0.38	0.536	0.051	[0.004, 0.174]
Ethnicity								
Non- Hispanic/Latinx	61.8%	38.2%	173					
Hispanic/ Latinx or other	76.9%	23.1%	65	1	4.13	0.042	0.142	[0.021, 0.268]
*N*	157	81						

### Common language results summary

#### What is the range of menstrual bleeding changes reported by regularly menstruating respondents after being administered the SARS-CoV-2 vaccine?

Respondents in our sample who menstruate regularly were about equally likely to have no bleeding changes after vaccination at all or to have heavier periods after vaccination. A much smaller proportion of people had lighter periods.

#### To what extent are nonmenstruating respondents reporting breakthrough bleeding after being administered the SARS-CoV-2 vaccine?

Greater than a third of respondents who used gender-affirming hormone treatments experienced breakthrough bleeding after vaccination. The majority of premenopausal respondents on long-acting reversible contraceptives (LARC) and the majority of postmenopausal respondents experienced breakthrough bleeding as well.

#### Are there trends among those with a changed bleeding pattern to help determine proximate mechanisms acting on the uterus?

Among regularly menstruating respondents, those who had heavier bleeding after vaccine were more likely to be older, be Hispanic/Latinx, have experienced fever and/or fatigue side effects, have a lighter typical menstrual flow, have been pregnant, and/or have given birth. Regularly menstruating people with endometriosis, menorrhagia, fibroids, and PCOS were slightly more likely to experience heavier bleeding. Among nonmenstruating premenopausal respondents, those with breakthrough bleeding after vaccine were more likely to have been pregnant and/or given birth. Last, among postmenopausal respondents, those with breakthrough bleeding after vaccine were more likely to be younger and/or be Hispanic/Latinx.

#### What else should I know about this research?

The nature of this survey means that we cannot compare the incidence of different experiences here with the general population (meaning, 40% of this sample having an experience does not mean that is the rate of that experience out in the world). The associations described here are not causal but provide evidence to better study these trends further. We emphasize that menstrual bleeding changes of this nature are generally not indicative of changes to fertility.

## DISCUSSION

We present initial summary statistics and descriptive analyses of changes to menstrual bleeding in a large and diverse sample of currently and formerly menstruating adults after SARS-CoV-2 vaccination. This is the very first characterization of postvaccine menstrual bleeding changes for a gender-diverse sample of pre- and postmenopausal people. We cannot estimate prevalence or incidence based on our methodological approach of this emergent phenomenon, and the associations reported here cannot establish causality. However, the trends we observe support hypothesis development for additional prospective studies in hemostatic and inflammatory changes to the endometrium after an acute immune response (Fig. 4).

In this first analysis, we focus on the heavier bleeding of currently menstruating and breakthrough bleeding of formerly menstruating people, which we define as an increased bleeding phenotype. The increased bleeding phenotype appeared to be the most common postvaccination change within our sample. Initial forays into our qualitative data suggest a widely variable experience of the increased bleeding phenotype, confounding a straightforward case definition. At this time, we suggest that, rather than a threshold quantity to define the increased bleeding phenotype, vaccinated people and providers instead consider menstrual changes in the context of what is typical for the vaccinated person. This definition is in line with recent changes to how heavy menstrual bleeding is described clinically (*50*), focusing more on lived experience and quality of life change than a particular quantity of blood loss.

Increased bleeding is often distressing, and it can (and often should) lead providers toward diagnostic procedures to assess its origins (*51*–*53*). This is especially true when it comes to breakthrough bleeding among formerly menstruating people, for whom this symptom can be an early sign of cancer. When possible side effects to a medical treatment are not shared with the clinical or patient population, it may lead to unnecessary, painful, and expensive diagnostic procedures. For example, several studies have now shown that epidurals likely increase the risk of heavy and breakthrough bleeding among regularly cycling and postmenopausal people, respectively (*54*, *55*). In one recent study, 17% of postmenopausal respondents reported breakthrough bleeding after injection, versus 7% from a control group. Of the 31 respondents who reported this bleeding to their physician, 13 had endometrial biopsies collected, and 2 had transvaginal ultrasounds. While all results were benign, endometrial biopsies are known to be painful and invasive procedures (*56*, *57*). Although these data have been reported in the literature for at least a decade, no patient-facing information about epidurals that we could find makes note of the risk of unexpected bleeding, which means that potentially unnecessary, expensive, painful diagnostic procedures may continue today.

Unexpected bleeding has other major and even life-threatening consequences. Trans men, trans masculine people, and masculine of center genderqueer people, many of whom suppress periods with a combination of LARC and masculinizing therapies, may find themselves suddenly navigating public bathrooms or workplaces while menstruating. Therefore, this unexpected bleeding runs the risk of psychological distress for those who experience gender dysphoria with menstruation and physical harm for people for whom managing menstruation in public is dangerous (*58*, *59*).

In addition to our finding of a substantial proportion of respondents experiencing some form of increased bleeding, we noticed some trends in who was more likely to have this phenotype. Among premenopausal 18- to 45-year-old respondents, those who were older and/or Hispanic or Latinx (using U.S. census demographic approaches) were more likely to report heavier bleeding after vaccine. Prior pregnancy and prior birth were also associated with a greater risk of heavier bleeding. Last, premenopausal menstruating respondents who were diagnosed with endometriosis, menorrhagia, fibroids, adenomyosis, and/or PCOS were more likely to report experiencing heavier bleeding after vaccine compared to those without any diagnosed reproductive condition. We also find that many respondents who had postvaccine changes did not have them until more than a week after inoculation, which extends beyond the typical 7 days of closely monitored adverse symptom reporting (i.e., solicited local and systemic adverse events) in vaccine trials [see (*40*, *41*)].

The responsiveness of menstrual cycles and bleeding patterns to external stressors is well known (*60*). Responsiveness to external stressors is one reason menstrual cycles are often thought of as reflecting overall health status, or a so-called “vital sign” in clinical practice (*61*–*63*). Thus, many people are attuned to menstrual cycles and take note of changes as potentially indicating other underlying health concerns. For many people, menstruation matters for reasons beyond current conceptive intentions: Menstruation relates to their experiences of gender and gender dysphoria, to their intuitive connections to bodily processes, and to their fears and embarrassments surrounding menstrual stigma (*58*, *64*, *65*). Therefore, unexpected and unplanned menstrual changes can cause concern, distress, or other negative responses, in addition to discomfort and physical pain.

Despite this, menstruation is seldom considered a variable during vaccine trials aside from determining last menstrual period as part of established protections against volunteers being or getting pregnant. The vast majority of research that has been conducted regarding reproductive and menstrual function centers around whether live and attenuated vaccines are safe to give to someone who is pregnant (*66*–*69*) or whether it affects fertility (*48*, *70*, *71*). The research that has been conducted on menstrual cycles specifically is often not able to establish a causal link, as the data are obtained through retrospective surveys or data mining (*72*, *73*) and randomized controlled trials often do not allow a mechanism for reporting these changes (*74*). Data mining and signal detection in the U.S. Vaccine Adverse Event Reporting System (VAERS) have resulted in the identification of several possible effects on menstruation that suggest that further research is needed (*72*, *73*); however, queries about changes in menstruation are still not a standard part of vaccine trials despite recent calls for more study (*75*).

Menstruation is an inflammatory and hemorrhagic event that must be resolved quickly to restore uterine function and prevent infection and continued hemorrhage (*14*, *76*). Disruption of the normal coagulation pathway of the endometrium may delay the repair mechanisms that allow menses to end quickly. A few of our findings suggest that vaccination is less likely to be affecting periods via ovarian hormone pathways, and more likely along these inflammatory pathways. For instance, we found little difference between respondents with spontaneous and hormonally contracepting cycles in the rate of postvaccine heavy menstrual flow. If changes in menstrual bleeding were due to vaccine-related disruption of ovarian hormones, we would expect that regularly menstruating people taking hormonal contraception would be far less likely to experience changes, as their cycles are largely regulated by exogenous hormones. We also found that a substantial proportion of formerly menstruating people, including postmenopausal participants with presumably dormant ovaries, experienced breakthrough bleeding. The greater presence of this increased bleeding phenotype among regularly cycling premenopausal respondents who were older and/or parous points to ways in which mature and established menstrual repair mechanisms may create a vulnerability to this short-term phenomenon. In addition, the greater proportion of people with certain reproductive conditions experiencing heavier bleeding after vaccine could also point to vulnerability among those with hyperproliferative and/or vascular/hemostatic conditions.

Data used in these analyses are unable to represent population prevalence of postvaccine menstrual changes. They may be biased toward those who noted some change in their own menstrual or bleeding experiences, particularly if that change was uncomfortable, painful, frightening, or concerning. That said, a substantial portion of the respondents who took part reported no menstrual change. Evidence suggests that people with other types of negative experiences are less, not more, likely to participate in surveys where they suspect that they will be expected to recount such material (*77*, *78*). A large number of qualitative responses alluded to the fact that people who are interested in science or cared about the research participated despite not having adverse menstrual experiences after vaccine.

Respondents in our sample were more likely to report fever (which was associated with heavier bleeding in our analyses) than participants in published vaccine safety and efficacy studies. Percentage comparisons between vaccines and studies, however, can be complicated by several factors, which include the age distribution of the sample, size of the sample, how the data are collected, and how the factor is defined to participants. Studies of multiple SARS-CoV-2 vaccine types indicated that younger participants reported higher incidence of fever than older participants (*40*, *41*, *79*–*82*). Vaccine safety studies that use a self-report measure of “feeling feverish” report higher percentages of affected participants compared to those that measured temperature (*80*, *82*, *83*). On the basis of literature across vaccine trial results, between 0.8 and 17.4% of participants reported having a fever after vaccination regardless of dose number, and between 2.5 and 71% experienced fatigue (*40*, *41*, *79*–*84*). Our survey asked participants to self-report fever, and so, we may have a significant portion of the sample who experienced elevated temperature that did not meet the clinical criterion. The other possibility is that those more likely to experience menstrual change are more likely to experience fever, and the potential selection bias of this sample may have therefore also increased the chances of fever appearing more frequently. It is not possible to tell from our data to what extent one or the other of these possibilities is more likely. Otherwise, the rate of other localized and systemic side effects reported in this sample were similar to that reported in vaccine trials (*40*–*42*). Awareness of selection bias is important to contextualize survey findings. That said, the large sample size for this survey allows us to describe the heterogeneity of symptoms and how these trends vary by respondent characteristics to identify avenues for future, hypothesis-driven mechanistic research.

An additional limitation of these analyses is due to the fact that our sample has a very high percentage of people who identify as white and as not Hispanic/Latinx. There are several potential causes for this, including that these data may reflect early trends in vaccination (*85*), it may be related to internet access (although the survey was tested for smartphone functionality), it may be related to participant trust in academic research, and/or it may be a function of how information about the survey was disseminated across social media and traditional media platforms. Social media allows for dissemination, but it also often creates insular communities due to differences in user demographics (*86*). Whatever the cause, we note the underrepresentation of Black, Indigenous, Latinx, and other respondents of color as a limitation in this research that seeks to understand menstrual experiences after vaccination. One of the ways we have sought to correct this underrepresentation is through the creation of a Spanish language version of the survey, which has only recently concluded.

Overall, our results align with other recent studies that show significant menstrual cycle responsiveness to SARS-CoV-2 vaccination. For example, a Norwegian cohort study found increased reports of heavier periods and longer menstrual bleeding after vaccination, which lasted for 2 to 3 months (*87*), and U.S.-based sample found longer cycle lengths after vaccination but no effect on bleeding duration (*88*). The only study published thus far that examined menstrual flow after vaccination had similar findings to ours, specifically that people using hormonal contraceptives were more likely to experience heavier bleeding after vaccination; however, they did not find an association with diagnosed reproductive conditions, although they note that their sample size might be too small and underpowered for this analysis (*89*). To the best of our knowledge, our work is the first to examine breakthrough bleeding after vaccination in either pre- or postmenopausal people. Furthermore, our large, gender-inclusive sample encompasses a broad age range, allowing us to more closely examine demographic trends and preexisting health and reproductive factors, which narrow down future avenues for further investigation.

Gaps in knowledge of how menstrual cycles respond to acute and chronic immune and inflammatory stressors can be understood as a form of ignorance, which is produced and reproduced based on structural, cultural, and political decisions (*90*). The data presented and discussed here highlight how anthropological mixed-methods research approaches that engage in listening rather than strictly pro forma hypothesis-driven research are necessary during emerging phenomena. Taking the time to listen and notice allows us to observe things that may not fit into our established narratives and to take responsibility for our role in knowledge dissemination as scientists (*91*, *92*). Furthermore, examination of the narratives and stories that we use to understand the world around us can illuminate the ways scientific narratives can shape and reproduce inequitable power structures of the world. Research that notices and attends to the experiences of people as well as our obligations and relations (*93*) is a necessary first step to building reciprocity (*94*) needed to restore trust and create transparency in science.

We have documented a phenotype of increased menstrual bleeding after COVID-19 vaccination across a diverse set of currently and formerly menstruating people. In doing so, we help provide evidence and context for clinicians regarding the validity of these experiences and we note future avenues of inquiry for researchers. Recognizing and attending to this emerging phenomenon of bleeding changes can help bolster trust between people who menstruate and medical providers, which is an area that has a long history of medical misogyny and gaslighting (*95*–*98*). Current and historic focus on fertility and reproduction in research and clinical trials is insufficient for addressing the changes in bleeding patterns that cause concern in many people. We urge other researchers and funding bodies to increase investment in understanding queer, trans, and nonbinary menstrual experiences, because there is a dearth of existing literature to understand the biosocial context of menstrual bleeding in these groups. Furthermore, we note that postmenopausal bleeding remains understudied. Mixed-methods and community-based participatory research to address questions that matter to those historically excluded from reproductive and menstruation science is needed to provide adequate and culturally and physically relevant care to these populations.

## MATERIALS AND METHODS

### Recruitment and survey information

This research was designated as exempt by the University of Illinois Institutional Review Board and Washington University in St. Louis Institutional Review Board. Data were collected and managed using REDCap (Research Electronic Data Capture) hosted at the University of Illinois at Urbana-Champaign (*99*, *100*). REDCap is a secure, web-based software platform designed to support data capture for research studies. The survey launched on 7 April 2021, and data for these preliminary results were downloaded on 29 June 2021 (approximately 12 weeks of data collection). The survey was initially announced on Twitter to recruit people who currently or previously menstruated and had been vaccinated (*101*, *102*), but it quickly propagated through multiple social media platforms. Media coverage (TV news, public radio, online journalism, print journalism, science blogs, etc.) of the study included links to the survey and provided widespread participant recruitment. In addition, many participants learned of the survey after performing an online search to investigate their own menstrual experiences and finding social media and/or news coverage of this project. Thus, the data collected by this survey represent extensive snowball sampling via many channels.

The survey included a mixture of multiple-choice and text entry questions about typical menstrual experiences (e.g., period flow, cycle length, bleeding duration, and common menstrual symptoms), menstrual experiences after each vaccine that make comparison to expected period symptoms (e.g., heavier/lighter/same), other menstrual symptoms, time between vaccine and menstrual side effects (multiple-choice question with ranges), reproductive history (e.g., history of pregnancy, parity, and history of postpartum hemorrhage), diagnoses of common reproductive conditions associated with altered menstrual bleeding patterns (e.g., endometriosis, adenomyosis, PCOS, menorrhagia, and fibroids), hormonal treatments [e.g., hormonal contraception, hormonal intrauterine devices (IUDs), and other treatments including gender-affirming hormones such as testosterone], and demographics. Cycle length and number of births were integer-validated text boxes, and most other questions were Yes/No, check boxes, or multiple-choice questions, which included an “other” or “not listed here” option to provide a text entry. The survey took approximately 15 to 20 min to complete. Additional details about study variables and the survey can be found in publicly archived supplemental information available at doi.org/10.17605/OSF.IO/6RVXK.

### Data cleaning

Data cleaning was performed on select text entries. Specifically, use of gender-affirming hormones, reasons for irregular menstruation or nonmenstruation, current pregnancy, IUD type, age, and postvaccine menstrual experience (e.g., breakthrough and menstrual flow) were coded from text responses or from existing survey questions. Common reasons for irregular menstruation or nonmenstruation were categorized by combining text entry responses with checkbox options added after the survey was live (i.e., using gender-affirming hormones, using LARCs, perimenopausal status, postmenopausal status, history of hysterectomy, current or recent lactation, and others). We screened for current pregnancy by evaluating respondent text responses regarding reasons for irregular or nonmenstrual status. No respondents in the analyzed sample reported being pregnant. IUD type was determined from text responses and categorized as hormonal, nonhormonal/copper, or unknown type. Respondents with age greater than 99 (*N* = 26) were manually adjusted on the basis of first two numbers entered (e.g., 323 was coded as 32) or by calculating the age from the birth year entered (e.g., 1990 was coded as 31).

Menstrual changes were coded on the basis of survey items across both vaccination doses. Flow change for regularly cycling people was coded on the basis of the dose 1 and dose 2 items assessing period flow with responses lighter, same, or heavier (see table S9). Because of the proportion of people experiencing heavy flow after at least one of the vaccines, we grouped regularly cycling individuals with any heavier flow into one condition (“heavier”), people who experienced no change in flow after either dose into the second condition (“no change”), and the remainder of people who experienced a combination of lighter and no change after their doses into a smaller third condition (“not heavier”). In total, 727 were missing dose 1 period flow information and 3031 were missing dose 2 period flow information (table S9). If information was missing at either dose, we treated it as pairwise missingness, so the respective menstrual change variable was missing. Nonmenstruating respondents were categorized as experiencing breakthrough bleeding if they reported spotting, a period, or other menstrual bleeding after either dose.

### Sample

At the time of downloading, 92,529 participants had completed the informed consent and submitted the survey. This included only unique email IDs with duplicate emails being sorted by time stamp, and the more recent time-stamped responses were retained (*N* = 205). Five individuals were removed for inappropriate and/or hostile responses. We removed participants under age 18 (*N* = 12). Responses missing more than 90% of survey items were removed (*N* = 11,999). From the remaining responses (*N* = 80,513), we retained only those that reported having not been diagnosed with COVID-19 (*N* = 65,241; removing *N* = 4494 with diagnosed COVID-19, 5761 with suspected but undiagnosed COVID-19, 4870 who were unsure about prior COVID-19, and 103 reporting other), as there is evidence that some people who contract COVID-19 have changes to menstrual bleeding (*34*). There were 42,097 who had received two vaccine doses, 19,161 who had not received a second dose, and 3983 who did not respond. Two-dose vaccinated individuals were restricted to those who submitted the survey at least 14 days after their second vaccination date (*N* = 35,660). Individuals who received only one dose (*N* = 23,144) were only included if they received the Johnson & Johnson vaccine and completed the survey at least 14 days after first dose vaccination date (*N* = 3469). In total, we removed 26,112 respondents who were not at least 14 days after full vaccination (i.e., 2 weeks after second dose for two-dose vaccines or 2 weeks after vaccination for single-dose vaccines). The final sample was 39,129 participants for general sample descriptive statistics.

We focused on the 35,660 individuals who received a two-dose SARS-CoV-2 vaccination for statistical analyses of menstrual changes and vaccine experiences. Of these respondents, baseline, prevaccine menstrual cycles were self-described as regular (*N* = 27,143), irregular (*N* = 4358), or absent (*N* = 4136), with 23 individuals not responding to this multiple-choice item and thus excluded from analyses beyond sample description. Analyses focus on conservatively defined subsamples based on self-reported typical prevaccine menstrual cycle status with additional restrictions to reduce the confounding influence of variables that likely affect menstrual cycles. We identified two major groups in the sample—those who regularly menstruate and those who do not currently menstruate but have in the past. Respondents who regularly menstruate are premenopausal people (ages 18 to 45) with either spontaneous menstrual cycles or hormonally contracepting cycles who still bleed regularly. Nonmenstruating respondents are premenopausal people (ages 18 to 45) on hormonal treatments that suppress menstruation (e.g., continuous use of hormonal contraceptives, LARC, and gender-affirming care such as testosterone) and postmenopausal people not on any hormonal treatments (ages 55 to 80; no period for at least 12 months). The majority of respondents who use gender-affirming care (242 of 267) specified testosterone. We included comparisons to those with diagnosed reproductive conditions generally (e.g., menorrhagia and endometriosis), as well as several specific reproductive conditions hypothesized to be relevant to inflammatory or hemostatic changes in the uterus. Further details can be found in the Supplementary Materials, and demographics are reported in tables S4 and S5. Briefly, we removed respondents who reported having a hysterectomy (*N* = 43), reported currently or recently lactating (*N* = 2498), and/or gave discrepant responses (e.g., self-reported period details did not align with self-reported menstrual group). The premenopausal sample was restricted to age below 45, and postmenopausal sample was restricted above age 55 due to the variability expected throughout perimenopause. People who reported having irregular menstrual cycles or were perimenopausal or at an uncertain menopause stage are not included in these analyses.

### Data analytic strategy

We started with descriptive statistics of the full sample of 39,129 fully vaccinated individuals grouped into age categories, omitting all second dose variables for the single-dose Johnson & Johnson vaccine. As this was an emerging phenomenon, we focus primarily on descriptive statistics and trends. We conducted preliminary analyses of associations between menstrual changes (i.e., flow change in menstruating respondents and breakthrough bleeding in nonmenstruation respondents) and race, ethnicity, vaccine type (restricted to the most common two-dose vaccines, Pfizer and Moderna), vaccine symptoms, typical period experience, reproductive history, and diagnosed reproductive conditions in preliminary univariate analysis of the sample groups using chi-square test of independence and using one-way analysis of variance (ANOVA) and *t* tests for age differences.

We then used a multivariate logistic regression based on the preliminary univariate associations. Specifically, the outcome was whether heavier flow (in regularly menstruation respondents) or breakthrough bleeding (in premenopausal nonmenstruating respondents) occurred after either dose of the vaccine, and the covariates were vaccine type, race, ethnicity, age, postvaccine adverse effects of fever and fatigue, diagnosis of a reproductive condition, contraceptive hormone use, history of bleeding during pregnancy, and history of postpartum hemorrhage. The nonmenstruating postmenopausal subgroup was too small for effective use of this statistical approach, and thus, we present stratified univariate analyses.

As the goal of this paper was to characterize the experiences of a wide range of people, we acknowledge the limitation of significance tests and primarily focus on effect size estimates and odds ratios. However, we also report and incorporate *P* values in our analyses and use them in combination with effect size estimates and confidence intervals when discussing results. Our analyses should be considered exploratory and descriptive to aid future hypothesis development to examine menstrual changes experienced following vaccines. All analyses were conducted in R (*103*). DescTools was used for chi-square test power analysis (*104*), rcompanion for Cramer’s *V* (*105*), questionr for odds ratio (*106*), and ggplot2 for figures (*107*). Additional details and supplements for the survey instrument are publicly archived: https://osf.io/6rvxk/?view_only=f91f1247658f49e3bbf59b2f6cfd3898.

## Notes

View/request a protocol for this paper from *Bio-protocol*.
