# Supplementary material for: Investigating trends in those who experience menstrual bleeding changes after SARS-CoV-2 vaccination
Source: Sci Adv. 2022 Jul 15;8(28):eabm7201. doi: 10.1126/sciadv.abm7201 (PMC9286513; doi:10.1126/sciadv.abm7201)
Supplement: Supplementary file 1 — Supplementary Text [file sciadv.abm7201_sm.pdf]

Science Advances
AAAS

Supplementary Materials for

Investigating trends in those who experience menstrual bleeding changes after SARS-CoV-2 vaccination

Katharine M. N. Lee *et al.*

Corresponding author: Kathryn B. H. Clancy, kclancy@illinois.edu

Sci. Adv. **8**, eabm7201 (2022)
DOI: 10.1126/sciadv.abm7201

The PDF file includes:

Supplementary Text
Legends for tables S1 to S9

Other Supplementary Material for this manuscript includes the following:

Tables S1 to S9

Supplementary Text

Study Variables

Flow change: The main outcome under investigation for regularly menstruating participants was the changes to period flow during the vaccination time. Two survey items addressed period flow, one for dose 1 and an identical item for dose 2, “After dose 1 [or 2] my period flow was…” with response options “lighter than usual”, “about the same”, or “heavier than usual”. For analysis, changes in bleeding heaviness were coded as “heavier” (based on reports of “heavier than usual” after either dose), “no change” (based on reporting “about the same” after both doses), and a third “heterogeneous no change or lighter” condition (based on a combination of reports of “about the same” or “lighter than usual” after either dose). Those that did not report flow information for either dose was missing flow change variable.

Length change: The second outcome variable for regularly menstruating individuals was change in period length or duration after vaccination. Two identical items measured the change in period length using the stem “After dose 1 [or 2] the length of my period was…” with responses “shorter”, “same”, or “longer”. We coded the following conditions: “longer” (based on reporting “longer” at either dose), “no change” (based on reporting “same” period length after both doses), “heterogeneous no change or shorter” (based on reporting a mixture of seeing no change or a shorter period length), or missing (anyone who failed to report length for either the dose 1 or the dose 2).

Breakthrough experienced: The outcome variable for non-menstruating individuals was the occurrence of breakthrough bleeding (defined as spotting, a period, and/or other menstrual bleeding) after vaccination following vaccine doses. In descriptive reporting, we examined whether breakthrough bleeding occurred after both doses, only dose 1, only dose 2, or did not occur at all. Participants who did not report any breakthrough bleeding are represented in the ‘None’ category. Breakthrough bleeding for statistical analysis was coded as 0= no breakthrough, 1= breakthrough bleeding.

Demographics: The demographic variables used were age, race, and ethnicity. A numerical entry text box asked age. A survey checkbox was used to address race: options were American Indian/Alaskan Native, Asian, Black or African American, Native Hawaiian or Pacific Islander, White, or other. A checkbox was also used for ethnicity with options of Non-Hispanic or Latinx, Hispanic or Latinx, and other. Race was coded as 0= White-only selected, 1=All other selections. Ethnicity was coded as 0=Non-Hispanic/Latinx-only selected, 1=All other selections. We describe all other selections as the diverse racial or diverse ethnic groups due to few participants in each category.

Usual menstrual experiences: Typical period flow and period length were assessed each with one item. Period flow was rated as ‘heavy’, ‘moderate’, ‘light’, ‘non-menstrual’, or ‘other’. Period length, or days with bleeding, were rated as ‘1-3 days long’, ‘3-5 days long’, ‘5-7 days long’, ‘7 or more days long’, ‘non-menstrual’, or ‘other’. The latter categories, non-menstrual and other, were excluded from subgroups as discrepant and from analysis, respectively.

Period symptoms: There were checkboxes to select period symptoms experienced following dose 1 [or 2]. The items were “gotten your period”, “experienced spotting”, “experienced other menstrual bleeding”, “experienced breast/chest soreness”, “experienced symptoms that you typically associate with your period (e.g., cramping, bloating)”, “other”, “I did not have any of these symptoms”.

Timing of period symptoms: Two survey items asked how long following dose 1 [or 2] before participants experienced the reported period symptoms. The options were 1-3 days, 4-7 days, 8-14 days, more than 14 days, or cycling at the time of the dose.

Vaccine symptoms: Participants responded to a single item asking whether they experienced any side effects following the first dose and, similarly, for the second dose. Subsequent items for vaccine symptoms following the first dose and second dose included checkboxes for arm-soreness, fever, fatigue, headache, nausea, or other. Fever and fatigue were identified as meaningful to compare alongside bleeding conditions, because they are more unrelated to a normal menstrual experience than headaches, for example. Fever was coded as 1 if participants said yes following either dose and 0 if it was not checked after either dose. Fatigue was, similarly, coded as fatigue following either or no fatigue after either dose.

Reproductive history: Participants answered whether they had ever been pregnant (coded 1=pregnancy, 0=no). They were asked, also, if they had ever given birth (1=parous, 0=no). A history of bleeding at either event were captured with the items, “Did you experience any vaginal bleeding during your pregnancies” and “Did you experience postpartum hemorrhage with any of your births”. Each subsequent question appeared contingent on having experienced the event of pregnancy or birth.

Reproductive conditions: A checkbox item assessed, “Have you ever been diagnosed with any of the following” with options heavy menstrual bleeding, abnormal uterine bleeding, menorrhagia, endometriosis, adenomyosis, fibroids, polycystic ovarian syndrome, and/or other condition you feel is relevant. The first three options were common descriptions given to describe a similar condition, which we refer singularly to as menorrhagia.

Additional details for subgroup definitions

1. Subgroups of Menstruating Sample.

The first group identified in the full sample were the individuals who reported not being diagnosed with any reproductive conditions (i.e., PCOS, endometriosis, menorrhagia or similar bleeding disorders, etc.), which likely affect people’s menstrual experience. People reported their typical menstrual cycle as regularly occurring (typically 20-40 day cycles that feel predictable), irregular or occasional (very far apart, not predictable, or both), or rarely or do not menstruate right now.

1.1. Regularly Cycling Individuals.

Those that reported regularly menstruating were restricted to a conservative group between the ages of 18 and 45 years-old, were not lactating in the last year, and had no history of hysterectomy.

1.1.1. *Spontaneous Regular Cyclers.* The conservative sample for regularly menstruating individuals that were not on any hormones (including birth control, thyroid treatment, other hormones) was 12,364. Discrepant responders were removed based on describing their usual period flow as 'non-menstrual' (n=4). Then we removed individuals that responded as not having a period after both doses (n=660). This subgroup of spontaneous regularly cycling comprised 11,700 participants. In the sample, 780 individuals reported having a copper, or non-hormonal IUD.

1.1.2. *Hormonally Contracepting Regular Cyclers.* The conservative sample for regularly menstruating individuals that were on hormonal contraceptives and/or other hormones was 4,185. Discrepant responders were removed based on describing their usual period flow or period length as 'non-menstrual' (n=25). Then we removed individuals that responded as not having a period after both doses (n=305). The subgroup comprised 3,855 participants that are hormonally contracepting and regularly cycling.

1.2. <u>Non-menstruating Individuals.</u>

Those that reported not menstruating for various reasons included 673 postmenopausal, 280 on gender affirming hormones/undergoing gender affirmative therapy, 1,911 on Long-Acting Reversible Contraceptives (LARC), 48 had hysterectomies (full or partial), 274 coded as being in an uncertain menopause stage, 463 peri-menopausal, 329 lactating recently, and 321 selected other responses not listed. Multiple reasons were able to be selected. We removed those that reported lactating recently (n=) or had a history of hysterectomy (n=48).

1.2.1. *Individuals undergoing Gender Affirmative Care.* The conservative sample of non-menstruating cycling individuals who described masculinizing therapy (e.g., testosterone) or reported undergoing gender affirming care was restricted to ages 18 to 45 years-old and, by definition, on hormones. Due to the use of some forms of birth control for gender affirmative treatment, we instead describe the categories of hormones the sample reported: 9 hormonal contraception only, 137 other hormones (e.g., testosterone) only, or 37 a mixture of both. In total, 27 were on hormonal contraceptives (i.e., birth control), 20 hormonal IUDs, 9 non-hormonal (copper) IUDs, and 174 reported other hormones. The gender-affirmative subgroup comprised 183 individuals.

1.2.2. *Individuals on Long-Acting Reversible Contraceptives.* The conservative sample of those on Long-acting Reversible Contraceptives (LARC) and non-menstruating cycling were restricted to ages between 18 and 45 years-old and, by definition, were on hormones. There are 41 individuals who are, also, represented in the gender affirmative group, so we removed them from the LARC subgroup. The hormones participants reported were grouped as 914 hormonal contraception only and 29 reported a mixture of other hormones and hormonal contraception. In total, 280 on hormonal contraceptives (i.e., birth control), 684 hormonal IUDs, 2 non-hormonal IUDs, and 29 on other hormonal treatments. The LARC subgroup was 943 individuals.

2. <u>Subgroups of Menstruating Sample diagnosed with Reproductive Conditions.</u>

The second group identified in the full sample were individuals that were diagnosed with one of a number of reproductive conditions (i.e., menorrhagia, endometriosis, adenomyosis, fibroids, PCOS, PPH, or other). There were 11,502 in the full sample that reported being diagnosed with at least one condition. The mean age was 36.28 years-old (SD=9.45 years; range from 18 to 77). Of this group, 7,774 were regularly menstruating, 1,949 were irregular, 1,768 were non-menstruating, and 11 did not respond. Two of the irregularly cycling participants reported having a full or partial hysterectomy along with 45 of the non-menstruating. So, we removed the individuals who reported having had a hysterectomy and those who were lactating recently, post-menopause, perimenopause, or in an uncertain menopause stage (n=10,105). Finally, we restricted the ages to 18 to 45 years-old in a conservative look at the menstrual changes to individuals diagnosed with reproductive conditions. The final sample number was 8,652.

2.1.1. *Individuals that are Spontaneous Regular Cyclers.* From the final reproductive conditions sample we first partitioned out individuals described as regularly cycling spontaneously. This subgroup included 4,013, which was further reduced by removing discrepant responses of 'non-menstrual' to either normal period flow and length (n=7). Anyone who reported not having had a period after both dose 1 and dose 2 were removed (n=221). Therefore, this spontaneous regularly cycling subgroup comprised N=3,785 participants. Non-hormonal IUDs were reported for 201 individuals in the subgroup.

2.1.2. *Hormonally Contracepting Individuals that are Regular Cyclers.* From the regularly cycling individuals in the reproductive conditions sample, we next focused on those hormonally contracepting (including those on other hormonal treatments or medications). This subgroup consisted of 2,218 individuals before we removed the discrepant responses (n=13) and those not having had a period (n=165). The final subgroup included 2,040 participants.

2.1.3. *Hormonally Contracepting Individuals that are Non-menstruating.* The portion of the sample diagnosed with a reproductive condition and described themselves as not menstruating was 942 individuals. Only 68 individuals were not on any hormonal treatments or medications, so we focused on hormonally contracepting individuals aged 18 to 45 diagnosed with at least one reproductive condition and described themselves as non-menstruating. The subgroup of non-menstruating individuals was 874. The majority of the sample described themselves as on LARC. And a small portion describes themselves as using gender-affirming hormones. Therefore, we focus on exclusive subgroups of LARC individuals and people on gender-affirming hormones.

2.1.3.1. Individuals Undergoing Gender Affirmative Care. The hormones reported by this group were 4 on hormonal contraceptives only, 57 on hormonal treatments only, and 26 on mixture of both: more specifically, 17 hormonal contraceptives, 14 hormonal IUDs, 2 copper IUDs, and 83 on other hormonal treatments. The final sample of people diagnosed with a reproductive

condition and non-menstruating undergoing gender-affirmative care were 87 individuals.

2.1.3.2. Individuals on Long-Acting Reversible Contraceptives. There are 24 individuals who are represented in the gender affirmative group, also, represented in the LARC group, so we removed them from LARC subgroup. The categories of hormones used were 549 on contraceptive hormones only, 2 on other hormones only (progestin), and 51 mixture of both: more specifically, 209 on hormonal contraceptives, 410 hormonal IUDs, 2 copper IUDs, and 53 on other hormonal treatments. The final sample for people diagnosed with a reproductive condition and non-menstruating on LARC were 602 individuals.

2.1.4. <u>Subgroups of Post-menopause Sample.</u>

2.1.4.1. Post-menopause without diagnosed reproductive conditions. The conservative sample for post-menopause individuals was restricted to ages 55 or older. Due to small sample numbers, we restricted to those not on any hormones (including birth control, thyroid treatment, other hormones). The subgroup post-menopause consisted of 117 individuals.

2.1.4.2. Post-menopause with diagnosed reproductive conditions. The conservative sample for post-menopause individuals who have a diagnosed reproductive condition was restricted to ages 55 or older and not on any hormones. The subgroup of post-menopause individuals with a diagnosed condition was 121 individuals.

Table S1. Full reporting of demographics and sample background. (separate file)

Expanded demographics and sample background. Full reporting for in-text Table 1.

Table S2. Menstrual changes and vaccine symptoms reported after each vaccine dose (full reporting). (separate file)

The reported symptoms and changes are grouped by age and by vaccine type. Dose 1 and dose 2 sample sizes differ because the Johnson&Johnson vaccine does not have a second dose, so participants do not report on any dose 2 survey items. Dose 1 and 2 period flow were used to calculate flow changes in regularly menstruating age groups. Period symptoms (had a period, spotting, and other menstrual bleeding) were used to calculate whether breakthrough occurred in non-menstruating age groups.

Table S3. Menstrual changes and vaccine symptoms reported after each vaccine dose (abbreviated reporting). (separate file)

The reported symptoms and changes as reported in Table S2 are grouped by age but not by vaccine type.

Table S4. Subsample demographics and background. (separate file)

The demographic reporting of the regularly cycling premenopausal respondents, the premenopausal non-menstruating respondents, and the postmenopausal respondents.

Table S5. Menstrual changes and vaccine symptoms reported after each vaccine dose. (separate file)

The reported symptoms and changes are displayed for the three sample groups: premenopausal regularly cycling, premenopausal non-menstruating, and postmenopausal respondents.

Table S6. Menstrual and medical history related to changes in flow across regularly cycling subgroups. (separate file)

These results refer to the univariate analyses of regularly cycling respondents for associations with flow change. Four subgroups are examined: spontaneous cycling with diagnosed reproductive conditions, the same without diagnosed reproductive conditions, hormonally contracepting with diagnosed reproductive conditions, and the same without diagnosed reproductive conditions.

Table S7. Proportion comparisons between premenopausal regular-cycling reproductive condition diagnoses and those without diagnosed reproductive conditions. (separate file)

These results refer to the univariate tests for associations between specific reproductive conditions versus no diagnosed condition and flow change.

Table S8. Vaccine and medical history related to breakthrough bleeding across pre-menopause non-menstruating groups. (separate file)

These results refer to the univariate analyses of premenopausal non-menstruating respondents for associations with breakthrough bleeding. The two groups are examined: those on LARC and those on gender affirming hormones.

Table S9. Contingency table of menstrual changes in all regularly cycling individuals (N=21,380).

The dose 1 period flow and dose 2 period flow contingency table displayed for all regularly cycling individuals.
